# High‐resolution bathymetry and water quality dataset from an impounded lake in Northeast Florida, USA

**DOI:** 10.1016/j.dib.2026.112838

**Published:** 2026-05-09

**Authors:** Daniele Pinton, Alberto Canestrelli, Nicole Dix, Lia Sansom

**Affiliations:** a University of Florida, Gainesville, FL 32611, USA; b Guana Tolomato Matanzas National Estuarine Research Reserve (GTMNERR), Florida Department of Environmental Protection, Ponte Vedra Beach, FL 32082, USA; c Biology Department, University of North Florida, Jacksonville, FL 32224, USA

**Keywords:** Autonomous surface vehicle, Estuarine monitoring, Eutrophication, Hydrodynamic modeling, HYCAT

## Abstract

Guana Lake, in Northeast Florida, is an impounded estuarine lake suffering from excess organic matter accumulation due to nutrient inputs from the watershed and restricted tidal flushing. This article presents a high-resolution dataset of bathymetry and water-quality parameters collected in Guana Lake compiled to support hydrodynamic and water quality modeling efforts. Data were acquired over four survey days in April-May 2023 using a YSI HYCAT autonomous surface vehicle equipped with a multibeam Acoustic Doppler Current Profiler (ADCP) and a multi-parameter water quality sonde. The dataset comprises thousands of point measurements of water depth, bed elevation, and water quality metrics (temperature, salinity, dissolved oxygen, pH, turbidity, and chlorophyll-a) at high-frequency across the ∼10 km length of the Lake. This data has been polished and is provided along with derived spatial maps of bathymetry and water quality distributions. The data are valuable for calibrating and validating numerical models of the Guana Lake system and similar estuaries, and they offer insights into managing water control structures to mitigate eutrophication. This dataset can be reused to investigate nutrient dynamics under different flushing scenarios, to test the performance of autonomous survey platforms in shallow vegetated waters, and to inform best practices for estuarine water quality management.

## Specifications Table

**Table:** 

Subject	Earth & Environmental Sciences
Specific subject area	Estuarine hydrodynamics and water quality management
Type of data	Field measurements: • ADCP (bathymetry) and EXO2 (water quality) data: text files (TXT format) • ProDSS data: spreadsheet file (CSV format) • Spatial maps/figures (processed images): ASCII files (ASC format) and PDF files
Data collection	Bathymetry, water depth, and water quality data were collected during field surveys using a YSI HYCAT autonomous surface vehicle. The HYCAT was equipped with a SonTek RiverSurveyor M9 ADCP for depth measurements and a YSI EXO2 multiparameter sonde for water quality [temperature, conductivity, salinity, dissolved oxygen (DO), pH, turbidity, chlorophyll-*a*, and fluorescent dissolved organic matter (fDOM)]. Data was recorded at high-frequency (i.e., 1 Hz for the ADCP and 4 Hz for the EXO2) and processed with MATLAB R2024b scripts. Validation of water quality data was manually recorded with a YSI ProDSS sonde.
Data source location	Guana Lake, Northeast Florida, USA (approximately 30°05’52” N, 81°20′21" W). Data processing and analysis were conducted at the University of Florida, Gainesville, FL, USA. The calibration of the sensors was conducted at the Guana-Tolomato-Matanzas National Estuarine Research Reserve (GTMNERR), Ponte Vedra Beach, FL, USA.
Data accessibility	Repository name: Zenodo Data identification number: 10.5281/zenodo.17853024, [1]. Direct URL to data: https://doi.org/10.5281/zenodo.17853024.
Related research article	Related technical report: Dix, N [2]. Related research article: Biondi et al. [3]. Two publications are in preparation [4,5].

## 1 Value of the Data

• *High-resolution spatial coverage*. This dataset provides an unprecedented high-resolution mapping of both bathymetry and water quality in Guana Lake (only “Lake” hereinafter), covering the entire lake with high spatial resolution measurements. Such comprehensive coverage is rarely available for shallow, vegetated estuarine systems and can improve the accuracy of hydrodynamic and water quality models for this environment.
• *Multiple parameters for integrated analysis*. The dataset includes synchronously measured physical and chemical parameters (water depth, bed elevation, temperature, salinity, dissolved oxygen, pH, turbidity, and chlorophyll), enabling multifaceted analysis of estuarine conditions. Researchers can reuse these data to explore relationships between bathymetric and water quality (e.g., how shallow areas correlate with high chlorophyll concentrations) and to validate sensor performance in the field.
• *Supports model development and calibration*. The data have been used to set up a Delft3D hydrodynamic and water quality model for Guana Lake (Biondi et al., in preparation). Other researchers, managers, or local users can reuse the bathymetric grid and boundary condition data to develop their own models or to test different scenarios of dam operation and tidal flushing. The availability of this dataset can save time in model calibration, as it provides real-world measurements to validate model outputs (such as simulated salinity distribution).
• *Informing management strategies*. Resource managers can leverage this dataset to better understand the spatial extent of organic matter accumulation and low water quality in the impounded Lake. The dataset captures baseline conditions under the management regime in April – May 2023. Therefore, it is valuable for evaluating the impact of potential remediation actions (like altered dam release schedules) on water levels and nutrient concentrations. The data can guide evidence-based decisions to reduce eutrophication in Guana Lake and similar managed estuaries.
• *Demonstration of autonomous survey technology.* The use of an autonomous surface vehicle [6] for shallow-water data collection is documented by this dataset. It provides a case study on the capabilities and limitations of such technology in dense aquatic vegetation and narrow channels. This is useful for other researchers or monitoring programs considering autonomous platforms for environmental data collection, as our dataset and accompanying notes highlight practical challenges (e.g., GPS signal loss, battery constraints, negative impacts of submerged vegetation) and successful strategies for data acquisition in difficult conditions. Data validation was performed by conducting simultaneous measurements using a second instrument and comparing the results for consistency.

## 2 Background

**Fig. 1:**
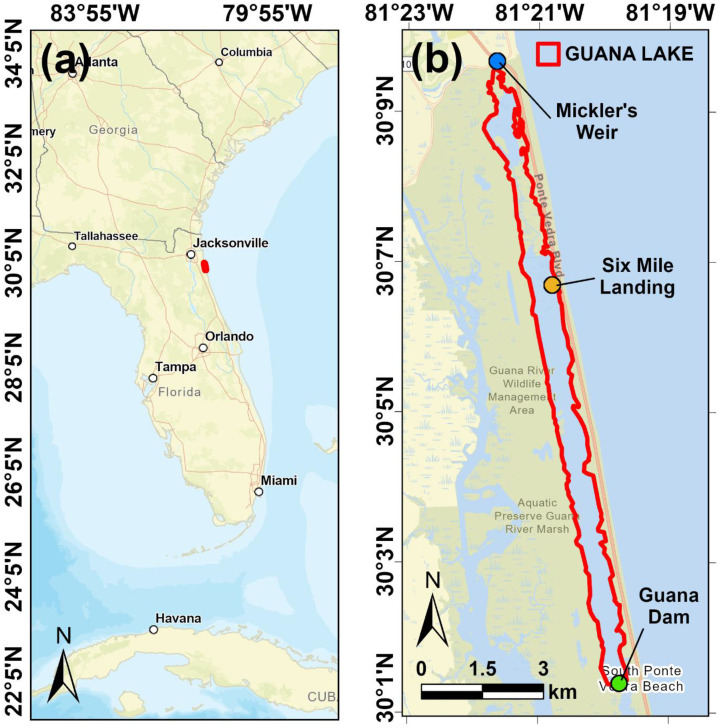
Map of the Guana Lake study area (b) in Northeast Florida (a). The boundaries of the impounded Lake are outlined in red. A water control structure at the south (Guana Dam, green dot) separates the Lake from the lower Guana River estuary, while a smaller dam at the north (Mickler’s Weir, blue dot) regulates inflow from the upper watershed. The Six Mile Landing boat ramp (orange dot) is a public access point on the east shore of the Lake.

Guana Lake is a dammed coastal waterbody located within the Guana-Tolomato-Matanzas National Estuarine Research Reserve (GTMNERR) in northeastern Florida (Fig. 1). Formed in 1957 by the construction of a dam that cut off tidal exchange with the estuary, the Lake has since experienced reduced flushing and ongoing freshwater input from an increasingly urbanized watershed. Recognizing its ecological and recreational importance, a public-private partnership was launched in 2017 to evaluate the condition of the Lake. This effort, led by GTMNERR, the Guana River Marsh Aquatic Preserve, and the Florida Fish and Wildlife Conservation Commission, identified nutrient enrichment, frequent algal blooms, and a strong north-to-south gradient in human influence [[7], [8], [9]].

To support water quality assessments and future restoration planning, an integrated field campaign was conducted between April and May 2023. Data collection combined vessel-mounted sensors and automated platforms to measure spatial patterns in depth, temperature, salinity, dissolved oxygen, nutrient concentrations, and algal biomass. These high-resolution measurements were post-processed and compiled into a georeferenced dataset, supporting ongoing numerical modeling of hydrologic reconnection scenarios. The dataset contributes to current analyses focused on internal nutrient loading, sedimentation processes, lake impoundment dynamics, and restoration design alternatives.

## 3 Data Description

The complete dataset includes georeferenced field measurements and processed data products. All data files are organized in a logical folder structure, published in a repository [1]. In summary, the dataset contains: (i) georeferenced bathymetric and water depth continuous sensor measurements, (ii) georeferenced water quality continuous sensor measurements, (iii) water depth and water quality validation manual measurements, and (iv) processed spatial data (gridded maps and images).

### 3.1 Bathymetry and water depth data

An exhaustive set of depth soundings was collected throughout Guana Lake using a SonTek RiverSurveyor M9 Acoustic Doppler Current Profiler (ADCP) mounted on the HYCAT autonomous survey vehicle. These measurements provided both real-time water depth and bed elevation relative to a reference datum. Raw data were logged at 1 Hz and included latitude, longitude, and instantaneous water depth (in meters). During post-processing, depth values were corrected for instrument offset and converted to bed elevation (in meters NAVD88) using contemporaneous water level readings (see Data Handling and Processing section). All vertical measurements are referenced to the North American Vertical Datum of 1988 (NAVD88), and horizontal coordinates are reported in the World Geodetic System 1984 (WGS 84 - EPSG:4326). Water depth and topographic data are organized as text files (TXT format). Timestamps are recorded in GMT. Each file includes the following columns:

• Date: The calendar day when the measurement was taken, formatted as MM/DD/YYYY.
• Time: The exact time the data point was recorded, formatted as HH:MM:SS (24-hour format).
• GPS Latitude: The latitude of the sampling location in decimal degrees, referenced to WGS 84 (EPSG:4326).
• GPS Longitude: The longitude of the sampling location in decimal degrees, also referenced to WGS 84 (EPSG:4326).
• Parameter Value: The final column of the water depth file provides water depth measurements in meters. The final column of the elevation file contains bed elevation values, also in meters, referenced to the North American Vertical Datum of 1988 (NAVD88).

**Fig. 2:**
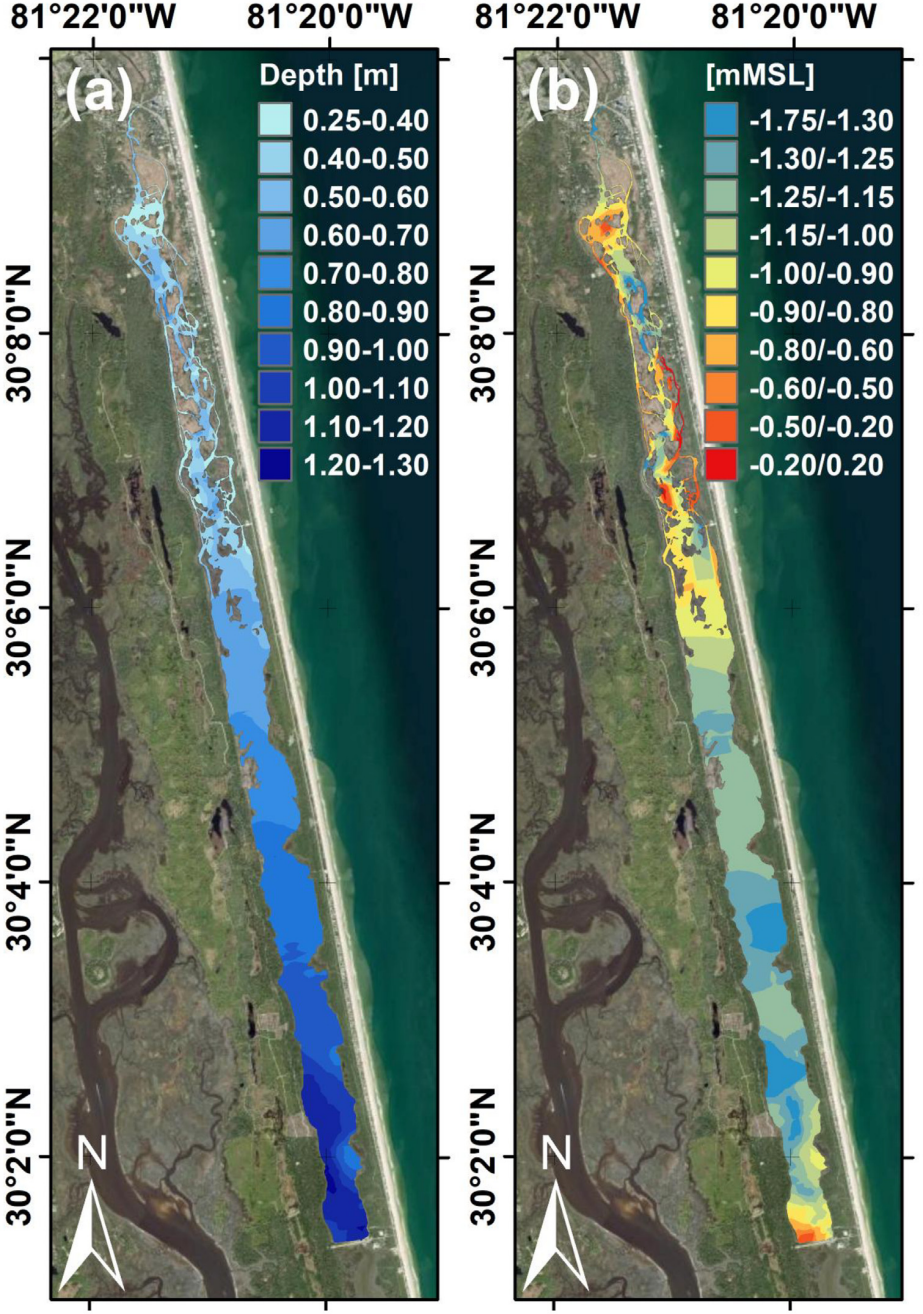
Spatial distribution of (a) water depth and (b) bathymetry in the Guana Lake. Spatial distributions are obtained by post-processing those collected in the Lake by using the ADCP on the HYCAT.

The georeferenced point data were interpolated onto a two-dimensional grid spanning the Lake to create a continuous bathymetric surface using the Inverse Distance Weighted (IDW) method (see “Data Handling and Processing” Section). A grid resolution of ∼10 meters was selected to accurately represent fine-scale bathymetric variability, including the narrow creeks in the northern portion of the Guana Lake. The water depth and bed elevation datasets were collected at high spatial resolution (1 Hz along the survey paths at a cruising speed of ∼2 knots (∼1 m/s)) within a short time window (late April–early May 2023), during stable hydrodynamic and meteorological conditions. The gridded data obtained from the IDW and shown in Fig. 2, do not represent time-averaged values but instantaneous spatial distributions reconstructed from the high-resolution survey measurements. Gridded data are provided in ASCII raster files (ASC format). Horizontal coordinates are in the World Geodetic System 1984 (WGS 84 - EPSG:4326). Fig. 2a shows the spatial distribution of water depth, while Fig. 2b displays the interpolated bathymetric map. These images are also provided in the repository as individual PDF files.

In the associated repository [1], data are included in the following files, contained in the “*txt.zip*”, “*ascii.zip*”, and “*pdf.zip*” folders:

• “*guana_lake_DP_2023_depth.txt*” contains the georeferenced water depth dataset.
• “*guana_lake_DP_2023_elevation.txt*” contains the georeferenced bed elevation dataset.
• “*guana_lake_DP_2023_depth.asc*” and “*guana_lake_DP_2023_elevation.asc*” contain the gridded water depth and bathymetry files.
• “*guana_lake_DP_2023_depth.pdf*” and “*guana_lake_DP_2023_elevation.pdf*” contain the maps of water depth and bathymetry.

### 3.2 Water quality data

A comprehensive suite of water quality parameters was also measured along the same transects used for the bathymetric survey, using a YSI/Xylem EXO2 multiparameter sensor mounted on the HYCAT autonomous survey vehicle. The sensor recorded high-frequency (i.e., 4 Hz) measurements of water temperature (°C), specific conductivity (µS/cm), salinity (ppt), dissolved oxygen (DO, in mg/L), pH, turbidity (FNU), chlorophyll-a (RFU), and fluorescent dissolved organic matter (fDOM, in RFU). All measurements are geo-referenced and time-stamped, ensuring spatial alignment with the bathymetric data. Each file includes the following columns:

• Date: The date the measurement was taken, formatted as MM/DD/YYYY.
• Time: The time of data collection, formatted as HH:MM:SS (24-hour format).
• GPS Latitude: The latitude of the sampling location in decimal degrees, referenced to WGS 84 (EPSG:4326).
• GPS Longitude: The longitude of the sampling location in decimal degrees, also referenced to WGS 84 (EPSG:4326).
• Parameter Value: The final column reports the recorded value of one specific water quality parameter listed above. Each file corresponds to a single parameter.

**Fig. 3:**
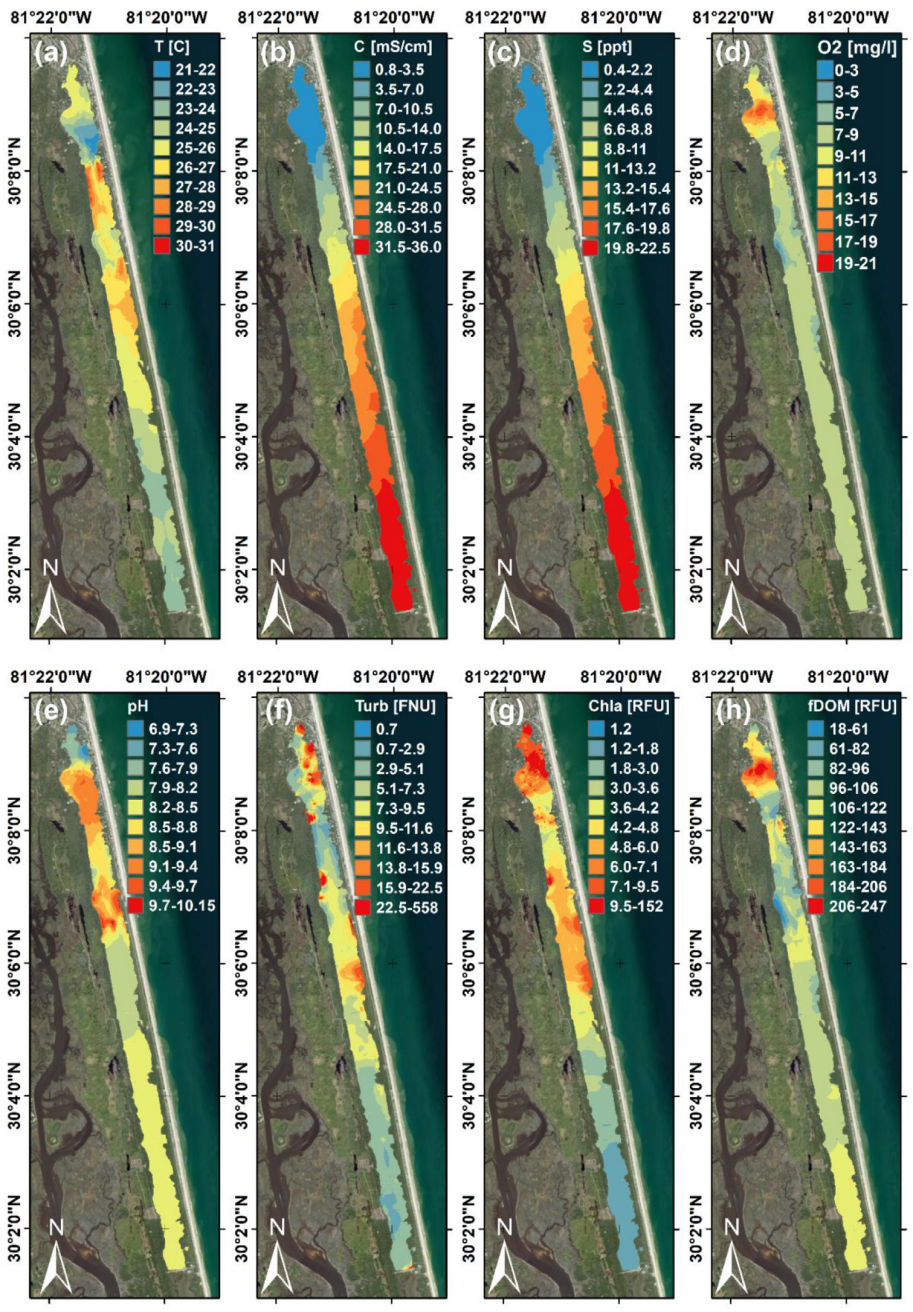
Spatial distribution of (a) water temperature, (b) specific conductivity, (c) salinity, (d) dissolved oxygen (DO), (e) pH, (f) turbidity, (g) chlorophyll-a, and (h) fDOM in the Guana Lake. Spatial distributions are obtained by post-processing those collected in the Lake by using the EXO2 on the HYCAT.

Raw sensor outputs were exported in TXT format and subjected to spatial and time referencing. This was done by assigning to each sample the time, latitude, and longitude of the four nearest points collected by the ADCP. Referenced datasets are provided in a text file (TXT format). These water quality datasets were interpolated onto a two-dimensional grid spanning the Lake surface, using the IDW method described in the “Data Handling and Processing” Section, to create a spatial distribution map for each parameter. Gridded data are provided in ASCII raster files (ASC format). Their spatial resolution is ∼10 m. These data are displayed in Fig. 3. The water quality datasets were collected at high spatial resolution (4 Hz along the survey paths at ∼2 knots (∼1 m/s)) within a short time window (late April–early May 2023), during stable conditions. The interpolated data shown in Fig. 3 represents instantaneous spatial distributions rather than time-averaged values. Horizontal coordinates are in the World Geodetic System 1984 (WGS 84 - EPSG:4326). Timestamps are recorded in GMT. These images are also provided in the repository as individual PDF files.

In the associated repository [1], data are included in the following files, contained in the “*txt.zip*”, “*ascii.zip*”, and “*pdf.zip*” folders:

• “*guana_lake_DP_2023_temperature.txt*” contains the georeferenced temperature dataset.
• *“guana_lake_DP_2023_conductivity.txt*” contains the georeferenced specific conductivity dataset.
• *“guana_lake_DP_2023_salinity.txt*” contains the georeferenced specific conductivity dataset.
• “*guana_lake_DP_2023_DO.txt*” contains the georeferenced dissolved oxygen dataset.
• “*guana_lake_DP_2023_pH.txt*” contains the georeferenced pH dataset.
• *“guana_lake_DP_2023_turbidity.txt*” contains the georeferenced turbidity dataset.
• “*guana_lake_DP_2023_chlorophyll.txt*” contains the georeferenced chlorophyll-a dataset.
• “*guana_lake_DP_2023_fDOM.txt*” contains the georeferenced fDOM dataset.
• “*guana_lake_DP_2023_temperature.asc,*” “*guana_lake_DP_2023_conductivity.asc,*” “*guana_lake_DP_2023_salinity.asc,*” “*guana_lake_DP_2023_DO.asc,*” “*guana_lake_DP_2023_pH.asc,*” “*guana_lake_DP_2023_turbidity.asc,*” “*guana_lake_DP_2023_chlorophyll.asc,*” and “*guana_lake_DP_2023_fDOM.asc*” contain the gridded temperature, specific conductivity, salinity, dissolved oxygen, pH, turbidity, chlorophyll-a, and fDOM files.
• “*guana_lake_DP_2023_temperature.pdf,*” “*guana_lake_DP_2023_conductivity.pdf,*” “*guana_lake_DP_2023_salinity.pdf,*” “*guana_lake_DP_2023_DO.pdf,*” “*guana_lake_DP_2023_pH.pdf,*” “*guana_lake_DP_2023_turbidity.pdf,*” “*guana_lake_DP_2023_chlorophyll.pdf,*” and “*guana_lake_DP_2023_fDOM.pdf*” contain the maps of temperature, specific conductivity, salinity, dissolved oxygen, pH, turbidity, chlorophyll-a, and fDOM.

### 3.3 Manually collected water quality data

**Fig. 4:**
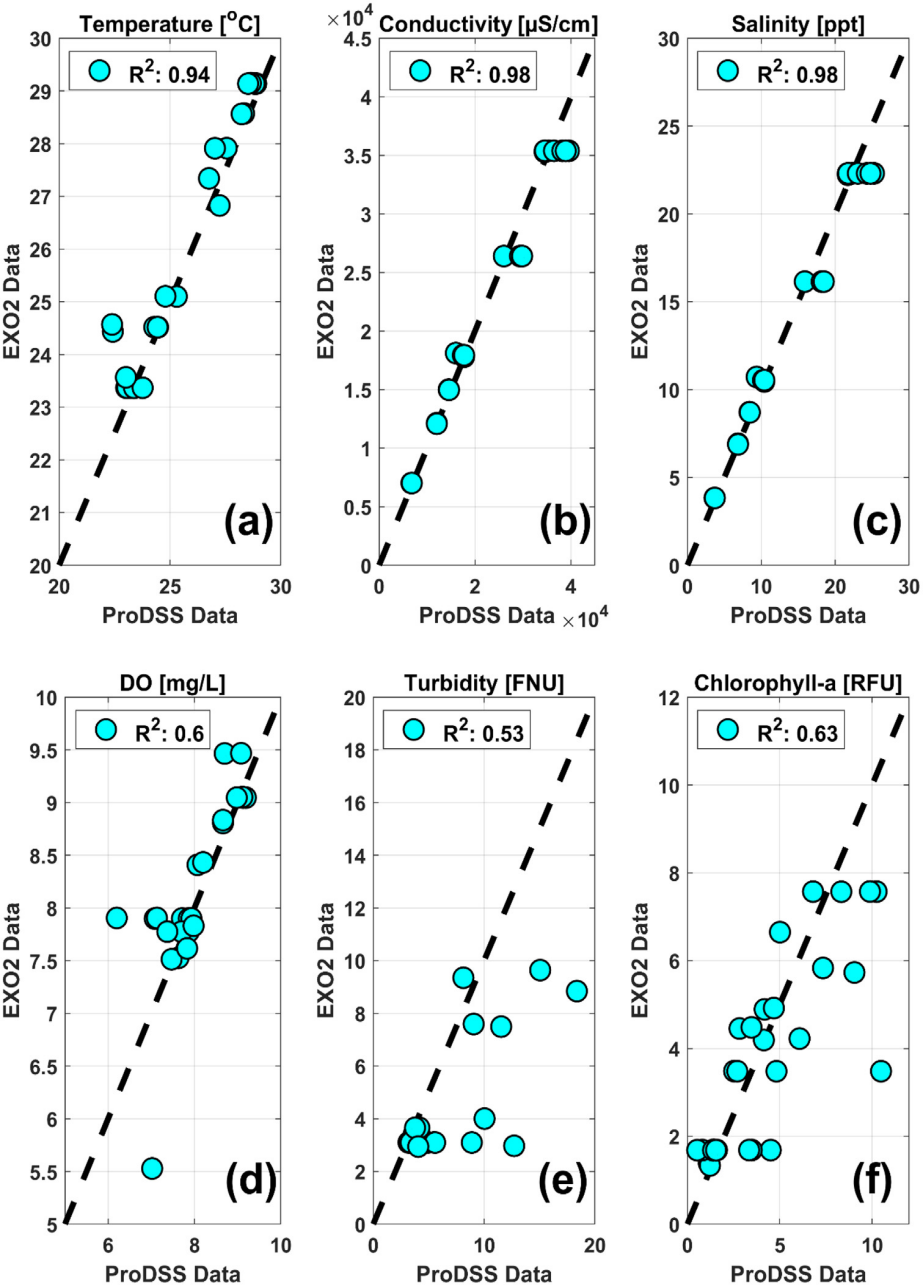
Comparison between the water quality data collected in the Guana Lake using the YSI ProDSS handheld sonde (on the abscissa axis) and the YSI EXO2 sonde mounted on the HYCAT (on the ordinate axis). The comparison is shown for (a) water temperature, (b) specific conductivity, (c) salinity, (d) dissolved oxygen (DO), (e) turbidity, and (f) chlorophyll-a.

To validate the autonomous HYCAT survey and support data quality assurance, discrete water quality measurements were collected using a YSI ProDSS handheld multiparameter sensor. These spot measurements were taken at selected surface locations across Guana Lake and included water temperature, salinity, specific conductivity, DO, turbidity, and chlorophyll-a. Each observation was georeferenced using the latitude, longitude, and time data collected by the ProDSS. The ProDSS readings served as independent validation for the continuous EXO2 sonde data and were used to confirm spatial trends observed in the high-frequency transect datasets. Validation results are provided in Fig. 4. The manually collected ProDSS data are compiled in a spreadsheet file (CSV format), including columns for sampling location, date/time, coordinates, and all recorded parameters. The file is named “*guana_lake_DP_2023_prodss.csv,*” and is contained in the “*prodss.zip*” folder in the associated repository [1].

### 3.4 Metadata

Each TXT and CSV data file in the repository is accompanied by metadata documentation describing its contents and format. The metadata documentation is contained in the “*metadata.zip*” folder. The data are in open text formats (i.e., TXT and CSV), ensuring that they can be accessed without proprietary software. In addition, all grids are in ASC format, which is accessible without proprietary software, and images derived from these datasets are in PDF format, which is vector-based and therefore preserves resolution and graphical quality regardless of scaling, while remaining accessible. Overall, this dataset captures a comprehensive snapshot of the environmental conditions of Guana Lake and is structured to be as user-friendly as possible for secondary analysis.

## 4 Experimental Design, Materials and Methods

### 4.1 Survey design

**Fig. 5:**
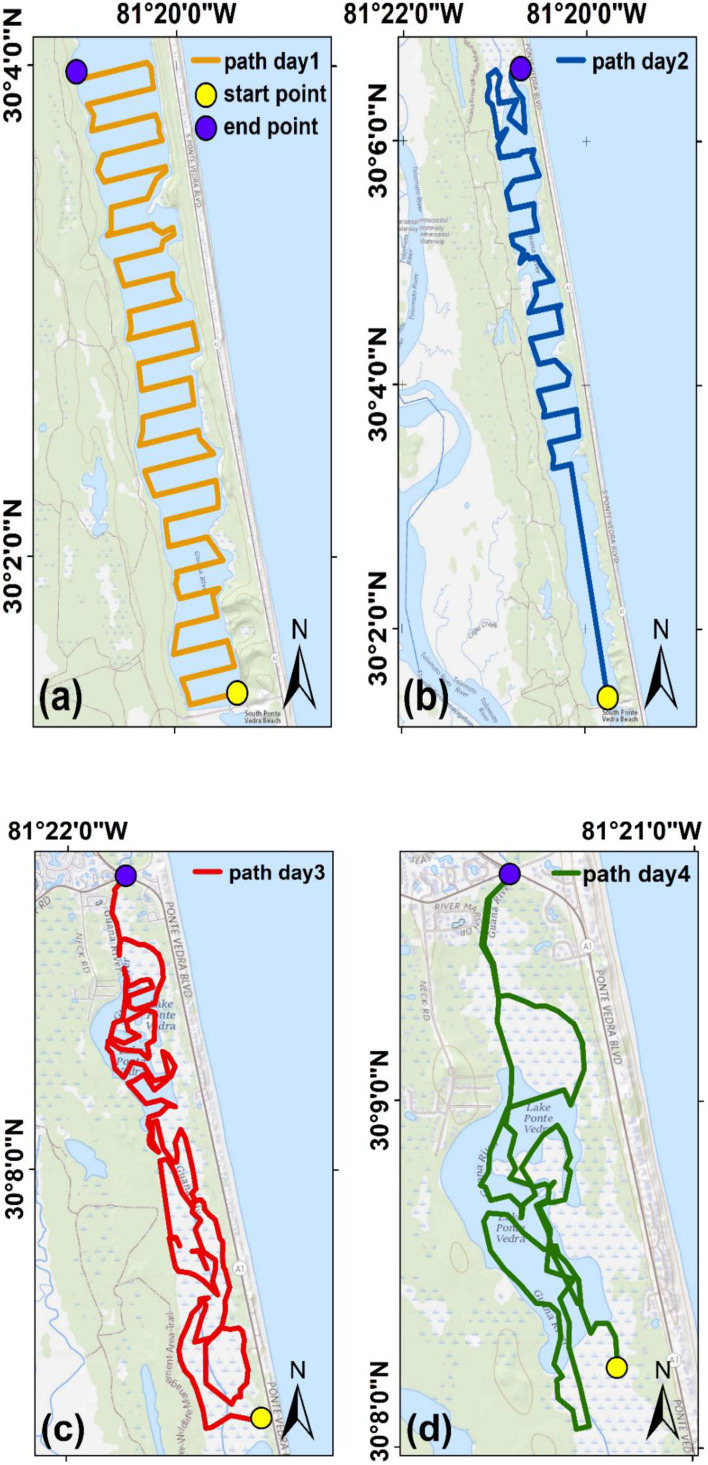
Planned survey tracks of the HYCAT autonomous surface vehicle during the four-day campaign in the Guana Lake (Days 1–4, Panels a–d). Yellow and violet dots are the start and end points of each daily survey, respectively.

The field data collection was planned to cover the entire Guana Lake with high spatial resolution while working within equipment endurance limits. The Lake was divided into four survey zones (south to north, Fig. 5), each of which could be sampled in approximately one working day (∼6-8 hours). Surveys were conducted on April 19, 20, 21, and May 2, 2023, under relatively calm weather and typical spring conditions. The first three days were used to survey the southern three-quarters of Guana Lake. Due to weather-related constraints, the remaining northern portion of the Lake was surveyed on May 2. Each day, the HYCAT autonomous surface vehicle was deployed to systematically traverse the designated zone. In the southern portion of Guana Lake, the navigation pattern combined a perimeter-hugging path along the shoreline with a series of zigzag transects across the open water areas. This approach was designed to maximize bathymetric and water quality coverage by capturing both shallow nearshore zones and deeper mid-lake regions, while ensuring adequate spatial resolution for bathymetric and water quality mapping. In contrast, the northern section of the Lake, characterized by a complex network of shallow minor creeks and deeper major channels between multiple islands, required a more intricate navigation pattern. Survey tracks in this area followed the natural geometry of the creeks to fully resolve bathymetric features and spatial variability in water quality. All navigation routes were pre-programmed using HYPACK (i.e., the software used to generate and initialize surveys with HYCAT) into the guidance system of the HYCAT using satellite imagery (obtained from Google Earth Pro and high-resolution NOAA imagery datasets, https://coast.noaa.gov/dataviewer/#/) to avoid obstacles and optimize coverage. However, in the narrower and shallower creeks of the northern Lake, the survey was manually conducted using the remote controller of the HYCAT. Manual operation was necessary to navigate around submerged vegetation, mudflats, exposed banks, and other potential obstructions, ensuring data collection without damaging the platform or compromising safety. The HYCAT was launched each morning from Six Mile Landing in the mid-lake or from a boat ramp near the Guana Dam (yellow dots in Fig. 5), depending on the zone being surveyed.

To support data validation and quality assurance, a handheld YSI ProDSS multiparameter sonde was used to collect discrete water quality measurements at selected surface locations across Guana Lake. These spot-check measurements were conducted each survey day from the support boat, following a planned sampling scheme that ensured representative coverage of both open water areas and vegetated creeks. At each sampling location, the ProDSS sonde was deployed to measure water temperature, specific conductivity, salinity, dissolved oxygen (DO), pH, turbidity, and chlorophyll-a. All measurements were taken at the surface (∼15–30 cm depth) after stabilization of sensor readings. Each observation was automatically georeferenced and time-stamped using the onboard GPS and clock of the ProDSS interface, eliminating the need for a separate handheld GPS unit. This ensured that all water quality data were accurately matched with their respective spatial and temporal coordinates. These measurements were used to verify the performance and consistency of the EXO2 sonde mounted on the HYCAT. Paired measurements at overlapping locations were compared to assess agreement between sensors. This redundancy ensured high confidence in the collected dataset and provided an independent validation of the autonomous survey results.

### 4.2 Instruments and operation

**Autonomous Surface Vehicle – HYCAT.** We used a SeaRobotics YSI HYCAT, a compact autonomous surface vehicle (ASV) designed for remote or pre-programmed survey operations in shallow water environments. The platform was equipped with two sensors: (i) a SonTek RiverSurveyor M9 Acoustic Doppler Current Profiler (ADCP) and (ii) a YSI/Xylem EXO2 multiparameter water quality sonde, each mounted on opposite sides of the HYCAT to minimize interference. Survey transects were conducted using a combination of autonomous and manual navigation modes. An operator followed the HYCAT at a safe distance (∼5–10 meters) in a support vessel, an airboat in the shallow, vegetated northern reaches of the Lake, and a mudboat in the deeper southern portion, driven by an expert pilot. This ensured constant radio communication with both the remote controller and the onboard laptop and allowed for quick retrieval if needed. The onboard GPS and electronic compass of the HYCAT guided its trajectory. The vehicle maintained a cruising speed of 2 knots (∼1 m/s) to ensure high-resolution, low-noise data collection, minimize turbulence around the sensors, and optimize battery life. The onboard battery provided up to ∼8 hours of operation at survey speed, aligning with the typical daily sampling window. In rare instances, the speed of the HYCAT was temporarily increased (up to 4 knots, i.e., ∼2 m/s) to maneuver around obstacles or overcome surface currents, though this was minimized to reduce data artifacts.

**SonTek RiverSurveyor M9 (ADCP)**. The ADCP operates by emitting acoustic pulses that measure water depth through bottom tracking of the returned echo. The SonTek RiverSurveyor M9 uses a suite of multi-frequency beams optimized for shallow environments, providing reliable depth measurements in water columns ranging from approximately 0.2 m (i.e., the blanking distance of the instrument) to several meters. For this survey, the M9 was configured to continuously record depth soundings directly beneath the HYCAT throughout each transect. Before each deployment, the instrument was calibrated and tested following manufacturer-recommended procedures documented in the sensor manual [1]. In addition to depth, the ADCP logged GPS position and time from the onboard navigation system of the HYCAT to ensure precise spatial referencing. All ADCP data were stored internally on the instrument and simultaneously transmitted via radio link to a laptop on the support vessel, providing real-time monitoring and an additional layer of data redundancy.

**YSI EXO2 multiparameter sonde.** This instrument was equipped with a suite of digital smart sensors to measure multiple water quality parameters. Specifically, the instrument included a Conductivity and Temperature Digital Smart Sensor (non-wiped, #599870) for temperature and specific conductivity (used to calculate salinity), a Dissolved Oxygen Optical Probe (#599100-01), a pH/ORP Digital Smart Sensor (unguarded, #577611), a Turbidity Optical Probe (#599101-01), a Smart Total Algae PE Sensor (#599103-01) that measures chlorophyll-a, and a fDOM Smart Sensor (#599104-01) that measures fDOM. All probes were calibrated in the lab at the GTMNERR before each survey according to the specifications of the manufacturer and the NOAA NERRS SWMP YSI/Xylem EXO Multi-Parameter Water Quality Monitoring SOP v2.1 [1], as well as the official EXO User Manual (archived on Pinton et al., 2025). After calibration, the sonde was mounted with sensors positioned approximately 0.15 m below the water surface while the HYCAT was in the water. It logged readings at 4 Hz, producing high-frequency and time-stamped data. Real-time telemetry allowed for live monitoring of the water quality data stream during each transect. This enabled the field team to detect anomalies such as temporary sensor fouling or drifting. All YSI EXO2 data were stored internally on the instrument and simultaneously transmitted via radio link to a laptop on the support vessel, providing real-time monitoring and an additional layer of data redundancy.

**YSI ProDSS multiparameter handheld sonde.** The YSI ProDSS is a portable, handheld multiparameter sonde used to collect discrete surface water quality measurements at selected locations across Guana Lake. The ProDSS was equipped with a suite of digital smart sensors similar to those used in the EXO2, except for the pH and fDOM sensors, which were excluded due to constraints on the number of sensor ports available. Specifically, the ProDSS configuration included a Conductivity and Temperature Digital Smart Sensor (#626902), used for measuring temperature and specific conductivity, from which salinity was derived, a Dissolved Oxygen Optical Probe (#626900), a Turbidity Optical Probe (#626901), and a Smart Total Algae PE Sensor (#626211), which measures chlorophyll-a. Measurements were collected near the surface (approximately 15–30 cm depth) with each data point automatically geo-referenced and time-stamped using the internal GPS and real-time clock of the ProDSS. Before each sampling session, all probes were calibrated at the GTMNERR lab following the guidelines of the manufacturer and in accordance with the NOAA NERRS SWMP YSI/Xylem EXO Multi-Parameter Water Quality Monitoring SOP v2.1 [1]. Following data collection, ProDSS data were downloaded, reviewed for quality control, and compiled into structured spreadsheets for integration and cross-comparison with EXO2 transect measurements.

### 4.3 Data handling and processing

After each survey, data were downloaded from all instruments (i.e., ADCP, EXO2, and ProDSS) and backed up. Initial quality checks involved removing obviously erroneous points (e.g., spurious depth readings when the ADCP pinged air if the HYCAT momentarily left the water, or sensor warm-up transients at the start of deployment). Additional filtering was applied using a statistical threshold of ±3 times the standard deviation of the measured data, with values exceeding this range flagged as outliers and removed. We also removed data exhibiting abrupt, nonphysical jumps relative to adjacent measurements, ensuring that only reliable observations were retained. Water depth measurements were converted to bed elevation by subtracting them from the elevation of the HYCAT platform, as recorded by the integrated GPS. The water level derived from the GPS was validated against measurements from a staff gauge located near the Guana Dam. On the first survey day, the only occasion when the HYCAT passed sufficiently close to the gauge, the GPS-derived water level (∼0.56 m NAVD88) closely matched the value recorded by the GTMNERR staff gauge (∼0.58 m NAVD88). We developed custom MATLAB (MathWorks R2024b) scripts to merge and analyze the datasets. The MATLAB scripts performed tasks such as interpolating scattered data onto a regular grid, using an Inverse Distance Weighted [10] interpolation for mapping. We also cross-validated the sonde data against the water samples (Fig. 4). The high $R^{2}$ values observed for water temperature (0.94), specific conductivity (0.98), and salinity (0.98), together with the moderate agreement for dissolved oxygen (0.60), turbidity (0.53), and chlorophyll-a (0.63), indicate that EXO2 measurements are consistent with ProDSS observations. This supports the use of the EXO2 sensor mounted on the HYCAT, or similar autonomous surface vehicles, to reliably characterize water-quality conditions in shallow and vegetated areas that are difficult to access with traditional surveys, enabling datasets suitable for numerical model calibration and validation, assessment of flushing dynamics, and development of improved estuarine water-quality management strategies [3,4,5]. The MATLAB scripts used for interpolating (i.e., generating the ASC files) and cross-validating the data are included in the repository for transparency (“*matlab.zip*” folder). The PDF maps were created from the shared ASC files in ArcGIS Pro (Version 3.2.0).

## Limitations

While comprehensive, this survey had some limitations:

• *GPS and communication dropouts.* The HYCAT occasionally experienced brief radio communication losses, which affected remote control and maneuverability but did not interrupt data logging**.** During these short dropouts, the vessel continued recording measurements, and no gaps in the dataset occurred.
• *Battery constraints.* The battery of the HYCAT lasts ∼8 hours at 2 knots, requiring slow navigation to complete each survey zone. Faster speeds would reduce battery life to ∼2 hours.
• *Speed constraints.* The escort airboat occasionally overheated, forcing brief pauses during which the HYCAT was halted.
• *Vegetation interference.* Dense submerged vegetation in northern creeks caused fouling of the ADCP and EXO2 sensors, leading to underestimated depths and temporary spikes in turbidity or chlorophyll-a from sediment resuspension. Although outliers are filtered, minor biases may remain. Data collected north of Six Mile Landing Point were acquired in densely vegetated areas and may be subject to increased uncertainty due to vegetation-induced interference.
• *Limited access to narrow channels.* Several upper creek branches were too narrow, shallow, or overgrown for the HYCAT or support boats to enter. Only partial measurements were possible, leaving small gaps in bathymetry and water quality coverage in these remote areas.

## Ethics Statement

The authors confirm that they have read and followed the ethical guidelines for publication in *Data in Brief*. This work did not involve any studies with human subjects or animals, and it did not utilize any data from social media platforms.

## CRediT Author Statement

**Daniele Pinton:** Conceptualization, Methodology, Investigation, Formal analysis, Data curation, Software, Visualization, Writing – original draft preparation, Writing – review & editing, Supervision, Project administration. **Alberto Canestrelli:** Conceptualization, Methodology, Project administration, Supervision, Writing – original draft preparation, Funding acquisition. **Nicole G. Dix:** Conceptualization, Resources, Project administration, Supervision, Writing – original draft preparation, Funding acquisition. **Lia Sansom:** Resources, Project administration, Funding acquisition.

## Data Availability

• ADCP (bathymetry) and EXO2 (water quality) data: text files (TXT format) • ProDSS data: spreadsheet file (CSV format) • Spatial maps/figures (processed images): ASCII files (ASC format) and PDF files.
